# Chronic immobilization stress occludes *in vivo* cortical activation in an animal model of panic induced by carbon dioxide inhalation

**DOI:** 10.3389/fnbeh.2014.00311

**Published:** 2014-09-16

**Authors:** Mohammed Mostafizur Rahman, Christian M. Kerskens, Sumantra Chattarji, Shane M. O'Mara

**Affiliations:** ^1^National Center for Biological Sciences, Tata Institute of Fundamental ResearchBangalore, India; ^2^Trinity College Institute of Neuroscience, Trinity College DublinDublin, Ireland

**Keywords:** carbon dioxide, hypercapnia, Magnetic Resonance Imaging, panic, somatosensory cortex, stress

## Abstract

Breathing high concentrations of carbon dioxide (CO_2_) can trigger panic and anxiety in humans. CO_2_ inhalation has been hypothesized to activate neural systems similar to those underlying fear learning, especially those involving the amygdala. Amygdala activity is also upregulated by stress. Recently, however, a separate pathway has been proposed for interoceptive panic and anxiety signals, as patients exhibited CO_2_-inhalation induced panic responses despite bilateral lesions of the amygdala. This paradoxical observation has raised the possibility that cortical circuits may underlie these responses. We sought to examine these divergent models by comparing *in vivo* brain activation in unstressed and chronically-stressed rats breathing CO_2_. Regional cerebral blood flow measurements using functional Magnetic Resonance Imaging (fMRI) in lightly-anaesthetized rats showed especially strong activation of the somatosensory cortex by CO_2_ inhalation in the unstressed group. Strikingly, prior exposure to chronic stress occluded this effect on cortical activity. This lends support to recent clinical observations and highlights the importance of looking beyond the traditional focus on limbic structures, such as the hippocampus and amygdala, to investigate a role for cortical areas in panic and anxiety in humans.

## Introduction

DSM-V defines panic attacks as “A discrete period of intense fear or discomfort, in which four (or more) of the enlisted symptoms (such as sweating, chest pain, paresthesias, trembling, nausea etc.), developed abruptly and reached a peak within 10 min” (American Psychiatric Association et al., 2013). Inhaling carbon dioxide (CO_2_) induces an emotional response similar to fear and panic attacks (Papp et al., 1997; Griez and Schruers, 1998). In healthy individuals inhaling high amounts of CO_2_ (20%) induces panic attacks (Forsyth et al., 2000). Panic disorder patients show panic response to even small doses of CO_2_ inhalation (Gorman et al., 1994). Patients with anxiety disorders or a family history of anxiety disorder are also more susceptible to show panic response to CO_2_ inhalation (Coryell, 1997). Inhaling hypercapnic gases, such as various concentrations of CO_2_, has been hypothesized to trigger a biological alarm system that has evolved to serve as a suffocation monitor (see Preter and Klein, 2008 for a review) (Klein, 1993; Preter and Klein, 2008). Earlier studies have tried to infer the neural circuit involved in this panic response. The hippocampus, amygdala and cortex (Sakai et al., 2005; Ziemann et al., 2009) have all been hypothesized to play a vital role in CO_2_-induced panic response. Some studies suggest that the panic response is similar to that caused by activation of the fear pathway (Gorman et al., 2000). Moreover, the amygdala can act as a chemosensor for pH changes, thereby regulating it's response to CO_2_ inhalation (Ziemann et al., 2009). Together these findings suggest a potential role for the amygdala in CO_2_-induced panic response. However, a recent study has reported CO_2_-induced panic response in human patients with amygdala lesions arising from Urbach-Wiethe disease (Feinstein et al., 2013). This hints at a potential parallel pathway for triggering intrinsic fear due to CO_2_-inhalation, possibly interoceptive in nature (see Critchley, 2005 for a review) (Critchley, 2005).

Stress-related psychiatric disorders have been shown to cause anxiety and enhanced fear responses in humans (Morgan III et al., 1995; Groome and Soureti, 2004). Since it has been hypothesized that panic response is similar to that caused by activation of the fear pathway, stress might show an influence on the panic response caused by CO_2_ inhalation. However, little is known about the interaction between stress and CO_2_-inhalation. Strengthening of the structural and physiological basis of synaptic connectivity in the amygdala has been implicated in chronic stress induced increase in anxiety and fear (Mitra et al., 2005; Suvrathan et al., 2014). However the fact that panic response due to CO_2_ inhalation involves the amygdala (Ziemann et al., 2009), but can also occur without amygdalar involvement (Feinstein et al., 2013), making it an intriguing case for further investigation. Therefore, we performed functional Magnetic Resonance Imaging (MRI) during the inhalation of CO_2_ in lightly-anaesthetized rats that were either unstressed or previously exposed to 10 days of chronic stress.

## Materials and methods

### Experimental animals

Twelve adult male Wistar rats (Trinity College Dublin BioResources Unit) weighing 270–300 g were used in the study. The animals were housed in groups of two. Animals were kept on a 12/12-h light/dark cycle and had access to water and a standard diet ad-libitum. All experiments were conducted in accordance with protocols approved by the Animal Ethics Committee of Trinity College, Dublin. These procedures were licensed by the Irish Department of Health and Children.

### Stress protocol

**Figure 1:**
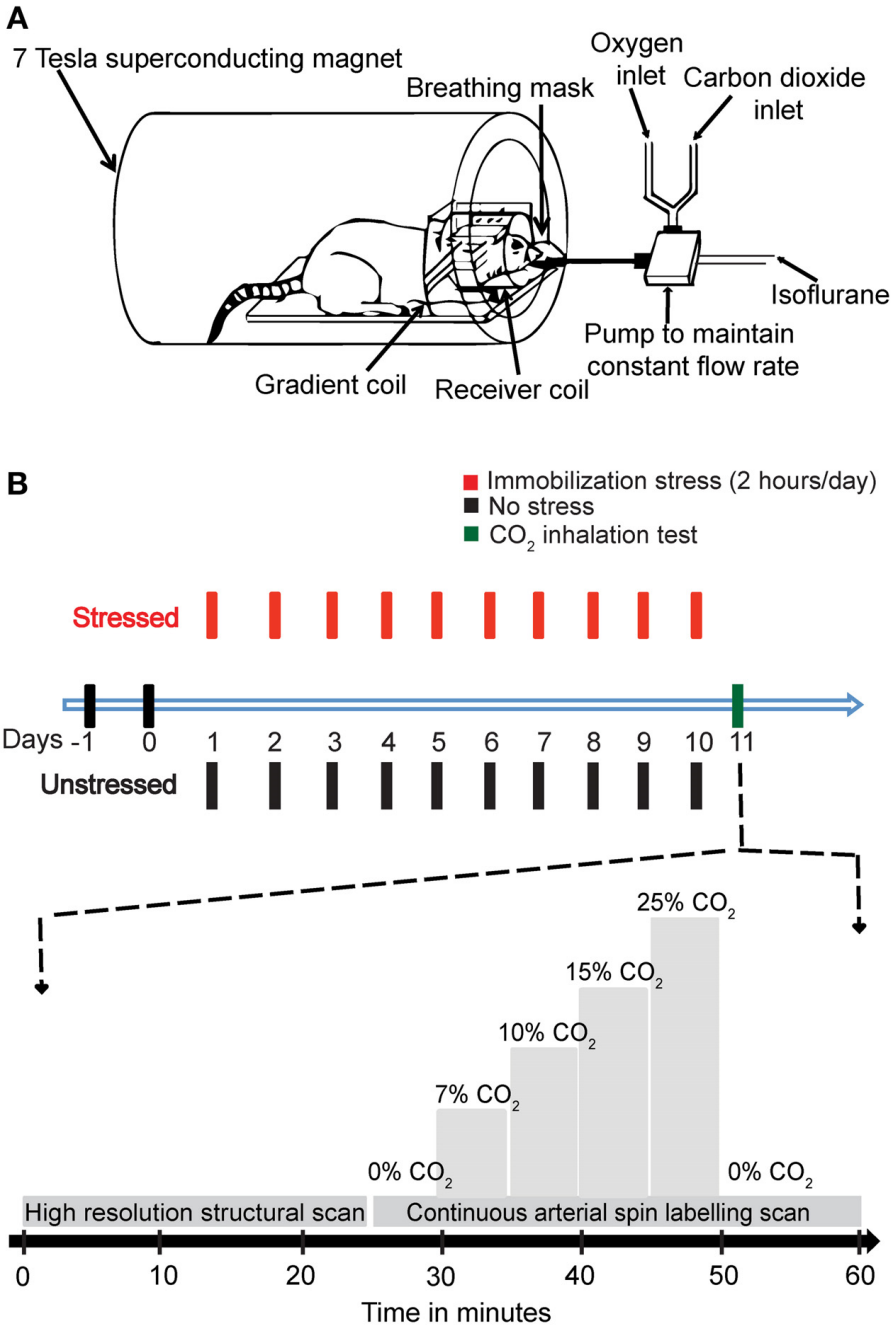
**The experimental design. (A)** A schematic representation of the experimental setup. **(B)** A diagrammatic representation of the experimental plan. The animals were handled for 2 days, and then the stressed rats were subjected to 2 h Immobilization Stress daily for a period of 10 days. On day 11, the rats were subjected to CO_2_ inhalation test along with Magnetic Resonance Imaging (MRI). A high-resolution structural scan was carried out first. Then a continuous arterial spin labeling (CASL) scan was carried out. During the scan for CASL the amount of CO_2_ in inhaled air was varied stepwise as indicated in the diagram.

The behavioral stress protocol has been described in several studies (Mitra et al., 2005). Briefly, rats were randomly assigned to experimental groups—stressed or unstressed. Rats in the stressed group were subjected to a chronic immobilization stress (CIS) paradigm. The CIS paradigm consisted of complete immobilization for 2 h per day (before noon) in rodent immobilization bags without access to either food or water, for 10 consecutive days. On the 11th day the rats were subjected to the CO_2_ inhalation test (Figure 1B).

### Animal preparation

All animals were anaesthetized with 5% isoflurane (Isoflo, Abbott, Queenboro, England) in oxygen (1 L/min) and maintained with 1.5–2% isoflurane. The level of anesthesia was regularly monitored throughout the procedure using the pedal withdrawal reflex to toe pinch. Tail vein blood was collected just after anesthetizing the rats as well as at the end of the experiment, to obtain blood gas samples. These samples were used to measure the concentration of carbon dioxide in blood before and after the experiment. We found no change in carbon dioxide concentration in the blood before and after the experiment. After the collection of samples the animals were placed prone in a Plexiglas cradle with a three point-fixation system (tooth-bar and ear pieces). Temperature was maintained constant at 37°C using a warming surface controlled by a water pump-driven temperature regulator. The respiration signal was monitored using custom hardware and software (SA Instruments Inc., Stony Brook, NY, USA). The animals were then placed in a 7T, 30 cm bore animal MRI system (Biospec 70/30, Bruker Biospin, Ettlingen, Germany) scanner with a circular polarized 1H rat brain RF coil (Bruker, BioSpin). A 7 cm diameter volume coil was used for transmission of the CASL and FLASH excitation pulses. Signal detection was performed using a surface coil.

### CO_2_-inhalation protocol

CO_2_ was mixed with oxygen (hyperoxic) and the percentage composition was maintained using partial pressure of the two gases. This mixture (1 L/min) replaced the oxygen supply. The amount of CO_2_ was maintained at the levels of 0, 7, 10, 15, and 20% in increasing order, with each concentration maintained for 5 min before changing the concentration. Finally, the amount of CO_2_ was decreased to 0% (Figure 1B).

### High resolution anatomical scans

**Figure 2:**
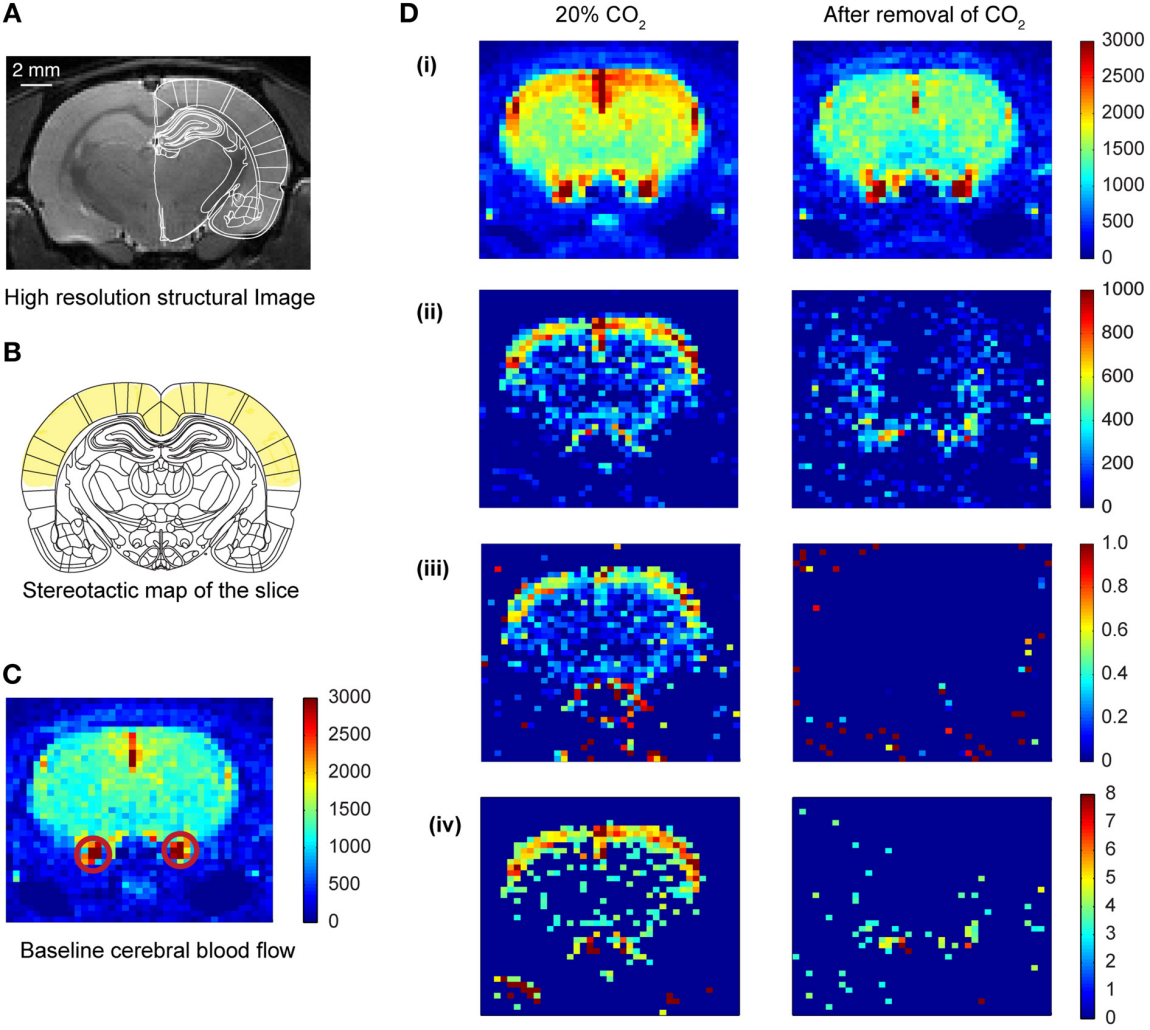
**Cerebral blood flow (CBF) pattern caused by inhalation of CO_2_. (A)** High resolution structural image of the slice acquired prior to CO_2_ inhalation. **(B)** Coronal plate from “The rat brain in stereotaxic coordinates” (Paxinos and Watson, 2005). Plate coordinates: −3.0 mm from bregma. All the imaging was done in the corresponding coronal slice plane. The region marked in yellow color represents mainly the motor cortex and somatosensory cortex. **(C)** Baseline CBF before the inhalation of CO_2_ of one of the animals. The colorbar represents the intensity of signal in a.u., which is directly proportional to regional cerebral blood volume (rCBV). The red circles show the carotid artery. **(D)** Transient CBF pattern caused by the inhalation of CO_2_. **(i)** The CBF during 20% CO_2_ inhalation and after removal of CO_2_. The colorbar represents values in a.u., which is directly proportional to rCBV. **(ii)** Subtracted images (activation—baseline) showing increased CBF only. **(iii)** Increase in CBF normalized to the baseline CBF. **(iv)** Z-score pixel wise (only pixels having z-score above 3 are represented here).

A rapid acquisition with relaxation enhancement (RARE) (Hennig et al., 1986) high resolution anatomical scan (slice thickness = 0.5 mm, *TE* = 36.7 ms, *TR* = 5.4595 s, FOV = 4 × 4 cm, image matrix = 256 × 256, RARE factor = 4) was performed and compared to a rat brain atlas in order to locate the slice with optimal somatosensory cortex, motor cortex, hippocampus and amygdala coverage (Figure 2A; Paxinos and Watson, 2005). This imaging slice location (thickness = 2.0 mm) was then used for the subsequent ASL sequence.

### Arterial spin labeling (ASL) MRI sequence

The ASL sequence consisted of a 5-s preparation interval containing the inversion pulse, followed by a snap shot fast low-angle shot (FLASH) image acquisition [echo time [TE] = 1.67 ms, repetition time [TR] = 5.0 ms, flip angle [FA] = 20°, bandwidth [BW] = 100 kHz, number of repetitions [NR] = 16, slice thickness = 2.0 mm, number of slices = 1, field of view [FOV] = 3.0 × 3.0 cm, matrix = 64 × 64, acquisition time = 5.082 s per repetition (Kerskens et al., 1996)]. Flow-induced fast adiabatic passage of inflowing inverted arterial spins was performed using a rectangular pulse. Inverted arterial spins then travel to the imaging plane (Dixon et al., 1986; Kelly et al., 2009). The inversion pulse radio frequency power (−22 dB) and offset frequency (−12 kHz) were determined to give optimal perfusion contrast by achieving inversion 2 cm proximal to the imaging plane. Control images with the offset frequency reversed (12 kHz), in which inflowing spins were left undisturbed, were also acquired, in an interleaved fashion. Eight repetitions of each image type were acquired for signal averaging using ParaVision 4.0 software (Bruker Biospin) for data reconstruction and analysis. Each individual 5-min segment of increasing CO_2_ concentration consisted of:

- Two minutes were given for the CO_2_ to reach the animal and get inhaled after increasing the CO_2_ concentration.
- Following this 2-min segment, 1.5 min were devoted to doing the scans.
- The final 1.5 min was provided for the animal to rest before the next increase in CO_2_ concentration was initiated.

Ten minutes were provided for the animals to recover back to baseline before scanning for the 0% CO_2_ concentration was done. Then the next increase in concentration was started.

### Qualitative ASL data analysis

Arterial spin images were exported to TIFF format from the 2dseq format (Bruker format, Bruker Biospin, Ettlingen, Germany) using ImageJ (US. National Institutes of Health, Bethesda, Maryland, USA) (Schneider et al., 2012). The exported images were subsequently processed and analyzed using custom written codes in MATLAB (MathWorks, Inc., USA). There were 4 scan sessions for each CO_2_ concentration. For each scan session 16 images were captured, 8 images with spin labeling and 8 corresponding control images. The two stacks were averaged and the average labeled image was subtracted from the average control image to get a result image for the corresponding scan (Griffin et al., 2010). The resulting images formed for each concentration were averaged to get the activation perfusion image at that concentration. The baseline perfusion image (0% concentration of CO_2_) was subtracted from the perfusion image at a certain concentration to obtain the change in perfusion induced by the CO_2_ inhalation. The change in perfusion image was normalized to the baseline image, in a pixel-by-pixel manner, to obtain the activation map due to CO_2_ inhalation (Figure 2D). The baseline perfusion and the perfusion image at a particular concentration were also used to calculate the z-score in a pixel-by-pixel manner. These were then threshold at z-score of 3 (Figure 2Div).

### Quantification of ASL data

The activation maps obtained by the method mentioned above were quantified to obtain the activation area and activation intensity. Intensity threshold was used in the baseline image to demarcate the brain slice. The number of pixels in the demarcated area was counted to calculate the total slice area. All the pixels in the demarcated area were used to calculate the root mean square noise (RMS noise) for each image, using the formula:

$$RMSnoise=\sqrt{\frac{\sum_{i=1}^{n}{\left(X_{i}-\frac{\sum_{i=1}^{n}X_{i}}{n}\right)}^{2}}{n}}$$

where *n* = total number of pixels, *X*_*i*_ is the intensity of each pixel.

**Figure 3:**
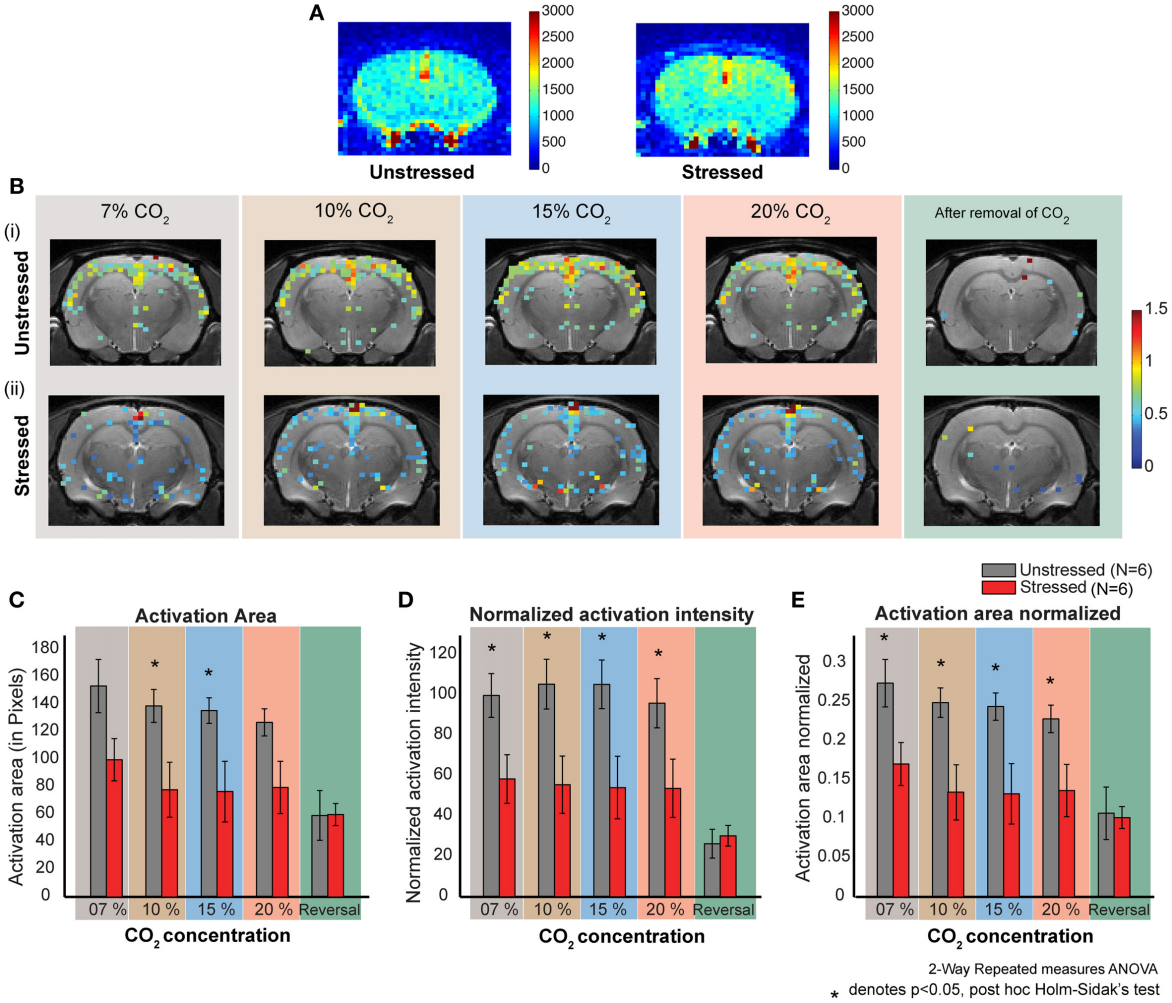
**CO_2_ induced dose dependent CBF in stressed and unstressed animals. (A)** Baseline CBF before the inhalation of CO_2_. The left image is from an unstressed animal and the right is from a stressed animal. The colorbar represents the intensity of signal in a.u., which is directly proportional to regional cerebral blood volume (rCBV). **(B)** Dose dependent response to CO_2_ inhalation. Each image represents the activation (increase in CBF normalized to the baseline) due to the inhalation of indicated percentage of CO_2_. **(i)** Represents images from an unstressed animal. **(ii)** Represents images from a stressed animal. **(C–E)** Quantification of response to CO_2_ inhalation in unstressed (*N* = 6) and stressed (*N* = 6) animals. **(D)** Normalized activation intensity (which is directly proportional to increase in cerebral blood flow normalized to baseline cerebral blood flow) in response to CO_2_ inhalation. There is a significant effect of stress (*p* = 0.0261) and CO2 inhalation (*p* < 0.0001). The response of unstressed and stressed rats is significantly different for all the concentrations CO_2_. **(C)** Activation area in response to CO_2_ inhalation. There is a significant effect of stress (*p* = 0.0404) and CO_2_ inhalation (*p* < 0.0001). The response of unstressed and stressed rats is significantly different for 10 % and 15% concentrations of CO_2_. **(E)** Activation area normalized to the total slice area in response to CO_2_ inhalation. There is a significant effect of stress (*p* = 0.0264) and CO_2_ inhalation (*p* < 0.0001). The response of unstressed and stressed rats is significantly different for all the concentrations of CO_2_.

Activation threshold was set at 2 × RMSnoise. Any pixel having intensity more than the threshold was considered an activated pixel. The normalized activation intensity was calculated by adding up the pixel intensities of all the activated pixels (Figure 3D). The total activation area was calculated by counting the number of activated pixels (Figure 3C). The activation intensity per pixel was obtained by dividing the normalized activation intensity with the activation area. The normalized activation area was calculated by dividing total activation area with the total slice area (Figure 3E).

### Statistical analysis

All statistical analysis was carried out using GraphPad prism (GraphPad software Inc., La Jolla, CA, USA). All results are presented as mean ± SEM unless otherwise stated. Statistical analysis was performed using repeated measures Two-Way ANOVA, unless otherwise stated. The *post-hoc* test performed was Holm–Sidak's test, unless otherwise mentioned. The criterion for statistical significance was *p* < 0.05.

## Results

In the current study, we used two groups of animals—control unstressed rats and rats that were subjected to 10 days of chronic immobilization stress (2 h/day) (Figure 1B top; Materials and Methods). The stressed animals showed a lack in bodyweight gain as compared to the unstressed animals (Supplementary Figure S1). This is in agreement with earlier studies confirming the efficacy of the chronic immobilization stress protocol (Vyas et al., 2002; Lakshminarasimhan and Chattarji, 2012). On the 11th day (i.e., 24 h after the end of the 10-day chronic stress paradigm), both groups of rats underwent fMRI (Figure 1A; Materials and Methods) during inhalation of varying levels of CO_2_ (Figure 1A bottom; Materials and Methods). To this end we focused on brain regions implicated in previous studies (Sakai et al., 2005; Ziemann et al., 2009; Gozzi et al., 2010). Therefore, we selected a brain slice that contained the amygdala, hippocampus and somatosensory cortex (Figure 2A). We first obtained a baseline perfusion map for the slice followed by a map during CO_2_ inhalation by the same rat (Figure 2C).

### Inhalation of CO_2_ triggers cortical activation

We observed an increase (15% relative to baseline) in perfusion (i.e., cerebral blood flow) across the entire brain sliceduringCO_2_ inhalation in unstressed rats (Figure 2D). Moreover, the increase in perfusion in the somatosensory cortex was greater (50% relative to baseline) than the whole-slice value. Thus, CO_2_-inhalation led to increased cortical activity. This activity pattern was transient and disappeared on removal of CO_2_ from the breathing gas mixture. Similar increases in perfusion have been reported due to increase in activity of neurons following forepaw stimulation (Griffin et al., 2010) suggesting that the increases we observe are within normal physiological ranges. In addition to this threshold-based analysis (Figures 2, 3) we also carried out an analysis based on z-scores. This analysis further confirms cortical activation CO_2_-inhalation (Supplementary Figure S2).

### Stressed rats show lower cortical activation arising from CO_2_ inhalation compared to unstressed rats

Both stressed and unstressed rats show increased cortical activation due to CO_2_-inhalation. However, the activation patterns are markedly different between the groups. The overall activation is less for the stressed group (Figure 3B, Supplementary Figure S2) and this pattern was evident across all CO_2_ concentrations tested (7, 10, 15, and 20%).

### Quantification of activation caused by CO_2_-inhalation

We carried out a more detailed quantification of the changes in activation pattern based on four parameters—normalized activation intensity, activation intensity per pixel, activation area and normalized activation area. An activation threshold was used to determine the activated pixels, which in turn were used to calculate these parameters (Materials and Methods). We used the normalized activation images for all the quantification, as these images give a more direct measure of cerebral blood flow (CBF) as compared to the z-score images (Griffin et al., 2010).

Normalized activation intensity is the sum of the normalized (to the baseline perfusion) intensities of all activated pixels. A high value for normalized activation intensity means high levels of cerebral blood flow. We see significantly higher levels of cerebral perfusion in unstressed rats compared to the stressed rats (Two-way repeated measures ANOVA, factor: stress, *p* < 0.05) (Figure 3D). The significant difference holds for all the CO_2_ concentrations used (*post-hoc* Holm–Sidak's multiple comparison test). Further, while unstressed rats show a significant decrease (*p* < 0.0001) in perfusion on removal of CO_2_ (*post-hoc* Sidak's multiple comparison test), stressed rats do not exhibit this decrease on removal of CO_2_. There were no dose-dependent differences in perfusion for either unstressed or stressed rats.

Activation intensity per pixel is the activation intensity normalized to the number of pixels in the slice. This is a measure of perfusion density, i.e., perfusion per pixel. We did not see any significant difference in perfusion density between unstressed and stressed rats (data not shown). However, perfusion density does decrease significantly after the removal of CO_2_ only in the unstressed rats (*p* < 0.05).

Activation area is the total brain area activated due to CO_2_ inhalation. We see a significantly higher activation area in unstressed, compared to stressed, rats (Two-Way repeated measures ANOVA, factor: stress *p* = 0.0404) for 10% and 15% CO_2_ (Figure 3C). There is no significant difference for the other concentrations (*post-hoc* Holm–Sidak's multiple comparison test). Further, unstressed rats show a significant decrease in activation area on removal of CO_2_, whereas the stressed rats do not show a significant decrease after CO_2_ removal (*post-hoc* Sidak's multiple comparison test). There was no dose-dependent difference in activation area for either unstressed or stressed rats.

Normalized activation area is the total area activated due to the inhalation of CO_2_ normalized to the total slice area. We see a significantly higher activation area in unstressed, compared to the stressed, rats (Two-way repeated measures ANOVA, factor: stress *p*-value = 0.0264) (Figure 3E). The significant difference holds for all the concentrations applied (*post-hoc* Holm–Sidak's multiple comparison test). The unstressed rats show a significant decrease in normalized activation area on removal of CO_2_, but the stressed rats do not show any significant decrease (*post-hoc* Sidak's multiple comparison test). There was no dose-dependent difference in normalized activation area for either unstressed or stressed rats.

Taken together, two patterns of difference emerge between control and stressed rats. First, inhalation of CO_2_ elicits an increase in all parameters, except perfusion density, in unstressed rats compared to their stressed counterparts. Second, removal of CO_2_ leads to a reduction in all the parameters in unstressed, but not stressed, rats. This might be due to the fact that the stressed animals do not show much activation due to CO_2_ in the first place.

## Discussion

### Cortical activation induced by CO_2_-inhalation

The present study is one of the first attempts to look at the consequences of two very different types of perturbations that trigger strong emotional effects and how they interact at the level of *in vivo* neural activity in the intact brain, normally, CO_2_ inhalation and how these effects interact with exposure to chronic stress. The most striking finding is that CO_2_ inhalation causes a distinct cortical activation. However we were not able to detect any activation in the amygdala.

We observed an increase in CBF (15% relative to the baseline) across the slice. This maybe due to a systemic response caused by the vasodilatory effect of CO_2_. Earlier studies have reported similar levels of CBF change (less than 10%) due to vasodilatory effects of CO_2_ inhalation in humans (Gambhir et al., 1997). However, the activation was the most pronounced in the somatosensory cortex (Figures 2B,D). Similar levels of increase in cerebral blood flow has been obtained due to increases in neuronal activity in earlier studies (Griffin et al., 2010). Therefore we observe activation of the somatosensory cortex due to CO_2_ inhalation. It is interesting to note that the somatosensory cortex has ASIC1 channels (Wemmie et al., 2003) which can act as chemosensor to CO_2_ and pH changes (Chung et al., 2010). Moreover, CO_2_-inhalation is known to trigger the trigeminal pathway (Wang et al., 2010), and human studies have reported activation of somatosensory cortex among other brain regions due to intranasal trigeminal function (Albrecht et al., 2010).

The primary somatosensory cortex has direct projections to the hypothalamus (Killackey and Sherman, 2003), which plays a pivotal role in maintaining body homeostasis (Williams et al., 2001) and control of the autonomic nervous system (Wang et al., 2007). Interestingly, the somatosensory cortex also projects to the nucleus raphé magnus and periaqueductal gray (Bragin et al., 1984; O'Hearn and Molliver, 1984). While the raphe nucleus is involved in both fear responses (Kiser et al., 1980) and respiratory control (Sessle et al., 1981), the periaqueductal gray mediates defensive behavior (Bittencourt et al., 2005; Schenberg et al., 2005) panic anxiety (Jenck et al., 1995; Schenberg et al., 2001) and suffocation (Kumar et al., 1997) among other autonomic responses. It has been hypothesized that the suffocation alarm system is located within the periaqueductal gray matter (Schimitel et al., 2012). Furthermore, key elements of the panic responses (e.g., increased heart rate, hyperventilation, increased perspiration, etc.), are controlled by the autonomic nervous system (Stein and Asmundson, 1994). Therefore, somatosensory cortex is well-positioned to serve as a possible candidate substrate to support, and perhaps initiate, the interoceptively-driven panic alarm triggered by CO_2_ inhalation. It also provides a possible candidate for an alternative pathway for fear and anxiety processing driven by interoceptive stimuli. In this connection, it is interesting to note that using a rat model of near-death experiences caused by cardiac arrest (Borjigin et al., 2013) have reported increase in cortical EEG gamma oscillations. Since prolonged hypercapnia also causes death, our findings highlight the need for further studies to determine the underlying oscillatory nature, if any, of the cortical signals associated with panic caused by CO_2_ inhalation.

### Occlusion of CO_2_-induced increase in cortical activity by prior exposure to stress

Panic attacks are more persistent in depression and stress-related psychiatric disorders (Nixon and Bryant, 2003), suggesting common or convergent pathways. The link between chronic immobilization and depression-like behavior has also been reported in rats and transgenic mouse models (Govindarajan et al., 2006; Suvrathan et al., 2010). Interestingly, we find that prior exposure to chronic stress occludes the effects of CO_2_ inhalation, which is manifested as a smaller change in cortical cerebral blood flow compared to unstressed animals (Figure 3B, Supplementary Figure S2). This might arise for two reasons. First, the somatosensory cortex in stressed subjects might be desensitized. The baseline activity might already be very high in stressed subjects, possibly approaching saturation, leaving little room for further enhancement by CO_2_ inhalation. Stress might also down regulate several signal transduction mechanisms implicated in CO_2_ detection (Sun et al., 2009; Wang et al., 2010). This could explain the relatively small increases induced by CO_2_ inhalation in stressed rats. Second, since we monitored cerebral blood flow, the lack of increase in activity might also imply a low cerebrovascular reserve capacity (Gambhir et al., 1997). However, our analysis shows that the decrease in activity results mainly from decreases in the active area rather than change per unit area (Figures 3C–E). This makes the first explanation more plausible.

The results presented here also raise the possibility that CO_2_ inhalation affects neural activity distributed across neural networks that have not been the traditional focus of rodent models of stress, anxiety and panic responses. The fact that we found no detectable changes within the amygdala does not rule out the possibility that projections from this structure to other brain areas, such as the somatosensory cortex, are not affected by the perturbations (stress and CO_2_ inhalation) used here. Indeed, growing evidence points to stress-induced modulation of connectivity between brain areas affected by repeated stress. For instance, a recent study (Ghosh et al., 2013) has shown that functional connectivity from the lateral amygdala to hippocampal area CA1 grows stronger, while directional coupling from area CA3 to CA1 within the hippocampus become weaker after chronic stress. Another study (Commins and O'Mara, 2000) found that stress facilitates the induction of long-term depression in the output regions of the hippocampal formation, thereby causing an effective decrease in connectivity. Future studies will be required to analyze how the spatiotemporal dynamics of interactions between cortical and subcortical areas give rise to brain activity changes seen following CO_2_ inhalation and consequent panic attacks.

Finally, our results using a rodent model are consistent with a recent report that, in humans, the internal threat signaled by CO_2_ can indeed elicit fear and panic despite the absence of an intact amygdala (Feinstein et al., 2013). This has led to the suggestion that CO_2_ might engage interoceptive afferent sensory pathways, which are distinct from pathways activated by exteroceptive stimuli. Furthermore, CO_2_ and pH-sensitive chemoreceptors are present in many brain areas outside the amygdala including the somatosensory cortex (Wemmie, 2011). Thus, the increased activity seen in the cortex may also be due to direct activation by CO_2_.

## Conclusion

A preponderance of prior evidence suggests that the panic response is mediated via an amygdalar fear pathway. However, a recent study suggests the presence of alternate and collateral pathways mediating fear and panic (Feinstein et al., 2013), independent of the amygdala under CO_2_ challenge. Here we imaged regions of the brain thought to play an important role in the panic response using CO_2_ challenge after stress induction. We found widespread changes cortical activation in unstressed animals, paradoxically not present in chronically-stressed animals, presumably because prior stress has already saturated cortical responsivity.

## Author contributions

Conceived and designed the experiments: Mohammed Mostafizur Rahman, Christian M. Kerskens, Sumantra Chattarji, and Shane M. O'Mara. Performed the experiments: Mohammed Mostafizur Rahman and Christian M. Kerskens. Analyzed the data: Mohammed Mostafizur Rahman. Contributed reagents/materials/analysis tools: Shane M. O'Mara. Wrote the paper: Mohammed Mostafizur Rahman, Sumantra Chattarji, and Shane M. O'Mara.

### Conflict of interest statement

The authors declare that the research was conducted in the absence of any commercial or financial relationships that could be construed as a potential conflict of interest.

## References

[B1] Albrecht J. Kopietz R. Frasnelli J. Wiesmann M. Hummel T. Lundström J. N. (2010). The neuronal correlates of intranasal trigeminal function-an ALE meta-analysis of human functional brain imaging data. Brain Res. Rev. 62, 183–196 10.1016/j.brainresrev.2009.11.001 19913573 PMC2822005

[B37] American Psychiatric Association, American Psychiatric Association, and DSM-5 Task Force. (2013). Diagnostic and Statistical Manual of Mental Disorders: DSM-5. Available online at: http://dsm.psychiatryonline.org/book.aspx?bookid=556 (Accessed: April 28, 2014).

[B2] Bittencourt A. S. Nakamura-Palacios E. M. Mauad H. Tufik S. Schenberg L. C. (2005). Organization of electrically and chemically evoked defensive behaviors within the deeper collicular layers as compared to the periaqueductal gray matter of the rat. Neuroscience 133, 873–892 10.1016/j.neuroscience.2005.03.012 15916856

[B3] Borjigin J. Lee U. Liu T. Pal D. Huff S. Klarr D. (2013). Surge of neurophysiological coherence and connectivity in the dying brain. Proc. Natl. Acad. Sci. U.S.A. 110, 14432–14437 10.1073/pnas.1308285110 23940340 PMC3761619

[B4] Bragin E. O. Yeliseeva Z. V. Vasilenko G. F. Meizerov E. E. Chuvin B. T. Durinyan R. A. (1984). Cortical projections to the periaqueductal grey in the cat: a retrograde horseradish peroxidase study. Neurosci. Lett. 51, 271–275 10.1016/0304-3940(84)90563-9 6514241

[B5] Chung W.-S. Farley J. M. Swenson A. Barnard J. M. Hamilton G. Chiposi R. (2010). Extracellular acidosis activates ASIC-like channels in freshly isolated cerebral artery smooth muscle cells. Am. J. Physiol. Cell Physiol. 298, C1198–C1208 10.1152/ajpcell.00511.2009 20181928 PMC2867380

[B6] Commins S. O'Mara S. M. (2000). Interactions between paired-pulse facilitation, low-frequency stimulation, and behavioral stress in the pathway from hippocampal area CA1 to the subiculum: dissociation of baseline synaptic transmission from paired-pulse facilitation and depression of the same pathway. Psychobiology 28, 1–11 10.3758/BF03330624

[B7] Coryell W. (1997). Hypersensitivity to carbon dioxide as a disease-specific trait marker. Biol. Psychiatry 41, 259–263 10.1016/S0006-3223(97)87457-4 9024948

[B8] Critchley H. D. (2005). Neural mechanisms of autonomic, affective, and cognitive integration. J. Comp. Neurol. 493, 154–166 10.1002/cne.20749 16254997

[B9] Dixon W. T. Du L. N. Faul D. D. Gado M. Rossnick S. (1986). Projection angiograms of blood labeled by adiabatic fast passage. Magn. Reson. Med. 3, 454–462 10.1002/mrm.1910030311 3724425

[B10] Feinstein J. S. Buzza C. Hurlemann R. Follmer R. L. Dahdaleh N. S. Coryell W. H. (2013). Fear and panic in humans with bilateral amygdala damage. Nat. Neurosci. 16, 270–272 10.1038/nn.3323 23377128 PMC3739474

[B11] Forsyth J. P. Eifert G. H. Canna M. A. (2000). Evoking analogue subtypes of panic attacks in a nonclinical population using carbon dioxide-enriched air. Behav. Res. Ther. 38, 559–572 10.1016/S0005-7967(99)00074-1 10846805

[B12] Gambhir S. Inao S. Tadokoro M. Nishino M. Ito K. Ishigaki T. (1997). Comparison of vasodilatory effect of carbon dioxide inhalation and intravenous acetazolamide on brain vasculature using positron emission tomography. Neurol. Res. 19, 139–144 9175142 10.1080/01616412.1997.11740787

[B13] Ghosh S. Laxmi T. R. Chattarji S. (2013). Functional connectivity from the amygdala to the hippocampus grows stronger after stress. J. Neurosci. 33, 7234–7244 10.1523/JNEUROSCI.0638-13.2013 23616532 PMC6619590

[B14] Gorman J. M. Kent J. M. Sullivan G. M. Coplan J. D. (2000). Neuroanatomical hypothesis of panic disorder, revised. Am. J. Psychiatry 157, 493–505 10.1176/appi.ajp.157.4.493 10739407

[B15] Gorman J. M. Papp L. A. Coplan J. D. Martinez J. M. Lennon S. Goetz R. R. (1994). Anxiogenic effects of CO2 and hyperventilation in patients with panic disorder. Am. J. Psychiatry 151, 547–553 8147452 10.1176/ajp.151.4.547

[B16] Govindarajan A. Rao B. S. S. Nair D. Trinh M. Mawjee N. Tonegawa S. (2006). Transgenic brain-derived neurotrophic factor expression causes both anxiogenic and antidepressant effects. Proc. Natl. Acad. Sci. U.S.A. 103, 13208–13213 10.1073/pnas.0605180103 16924103 PMC1559778

[B17] Gozzi A. Jain A. Giovannelli A. Giovanelli A. Bertollini C. Crestan V. (2010). A neural switch for active and passive fear. Neuron 67, 656–666 10.1016/j.neuron.2010.07.008 20797541

[B18] Griez E. Schruers K. (1998). Experimental pathophysiology of panic. J. Psychosom. Res. 45, 493–503 10.1016/S0022-3999(98)00027-0 9859852

[B19] Griffin K. M. Blau C. W. Kelly M. E. O'Herlihy C. O'Connell P. R. Jones J. F. X. (2010). Propofol allows precise quantitative arterial spin labelling functional magnetic resonance imaging in the rat. Neuroimage 51, 1395–1404 10.1016/j.neuroimage.2010.03.024 20304075

[B20] Groome D. Soureti A. (2004). Post-traumatic stress disorder and anxiety symptoms in children exposed to the 1999 Greek earthquake. Br. J. Psychol. 95, 387–397 10.1348/0007126041528149 15296542

[B21] Hennig J. Nauerth A. Friedburg H. (1986). RARE imaging: a fast imaging method for clinical MR. Magn. Reson. Med. 3, 823–833 10.1002/mrm.1910030602 3821461

[B22] Jenck F. Moreau J.-L. Martin J. R. (1995). Dorsal periaqueductal gray-induced aversion as a simulation of panic anxiety: elements of face and predictive validity. Psychiatry Res. 57, 181–191 10.1016/0165-1781(95)02673-K 7480384

[B23] Kelly M. E. Blau C. W. Kerskens C. M. (2009). Bolus-tracking arterial spin labelling: theoretical and experimental results. Phys. Med. Biol. 54, 1235–1251 10.1088/0031-9155/54/5/009 19182324

[B24] Kerskens C. M. Hoehn-Berlage M. Schmitz B. Busch E. Bock C. Gyngell M. L. (1996). Ultrafast perfusion-weighted MRI of functional brain activation in rats during forepaw stimulation: comparison with T2 -weighted MRI. NMR Biomed. 9, 20–23 8842029 10.1002/(SICI)1099-1492(199602)9:1<20::AID-NBM381>3.0.CO;2-R

[B25] Killackey H. P. Sherman S. M. (2003). Corticothalamic projections from the rat primary somatosensory cortex. J. Neurosci. 23, 7381–7384 12917373 10.1523/JNEUROSCI.23-19-07381.2003 PMC6740432

[B26] Kiser R. S. Brown C. A. Sanghera M. K. German D. C. (1980). Dorsal raphe nucleus stimulation reduces centrally-elicited fearlike behavior. Brain Res. 191, 265–272 10.1016/0006-8993(80)90331-5 7378757

[B27] Klein D. F. (1993). False suffocation alarms, spontaneous panics, and related conditions. An integrative hypothesis. Arch. Gen. Psychiatry 50, 306–317 10.1001/archpsyc.1993.01820160076009 8466392

[B28] Kumar K. Toth C. Nath R. K. (1997). Deep brain stimulation for intractable pain: a 15-year experience. Neurosurgery 40, 736–746 discussion: 746–747. 10.1097/00006123-199704000-00015 9092847

[B29] Lakshminarasimhan H. Chattarji S. (2012). Stress leads to contrasting effects on the levels of brain derived neurotrophic factor in the hippocampus and amygdala. PLoS ONE 7:e30481 10.1371/journal.pone.0030481 22272355 PMC3260293

[B30] Mitra R. Jadhav S. McEwen B. S. Vyas A. Chattarji S. (2005). Stress duration modulates the spatiotemporal patterns of spine formation in the basolateral amygdala. Proc. Natl. Acad. Sci. U.S.A. 102, 9371–9376 10.1073/pnas.0504011102 15967994 PMC1166638

[B31] Morgan C. A. III Grillon C. Southwick S. M. Davis M. Charney D. S. (1995). Fear-potentiated startle in posttraumatic stress disorder. Biol. Psychiatry 38, 378–385 10.1016/0006-3223(94)00321-S 8547457

[B32] Nixon R. D. V. Bryant R. A. (2003). Peritraumatic and persistent panic attacks in acute stress disorder. Behav. Res. Ther. 41, 1237–1242 10.1016/S0005-7967(03)00150-5 12971943

[B33] O'Hearn E. Molliver M. E. (1984). Organization of raphe-cortical projections in rat: a quantitative retrograde study. Brain Res. Bull. 13, 709–726 6099744 10.1016/0361-9230(84)90232-6

[B34] Papp L. A. Martinez J. M. Klein D. F. Coplan J. D. Norman R. G. Cole R. (1997). Respiratory psychophysiology of panic disorder: three respiratory challenges in 98 subjects. Am. J. Psychiatry 154, 1557–1565 9356564 10.1176/ajp.154.11.1557

[B35] Paxinos G. Watson C. (2005). The Rat Brain in Stereotaxic Coordinates. Burlington, MA: Elsevier Academic Press

[B36] Preter M. Klein D. F. (2008). Panic, suffocation false alarms, separation anxiety and endogenous opioids. Prog. Neuropsychopharmacol. Biol. Psychiatry 32, 603–612 10.1016/j.pnpbp.2007.07.029 17765379 PMC2325919

[B38] Sakai Y. Kumano H. Nishikawa M. Sakano Y. Kaiya H. Imabayashi E. (2005). Cerebral glucose metabolism associated with a fear network in panic disorder. Neuroreport 16, 927–931 10.1097/00001756-200506210-00010 15931063

[B39] Schenberg L. C. Bittencourt A. S. Sudré E. C. M. Vargas L. C. (2001). Modeling panic attacks. Neurosci. Biobehav. Rev. 25, 647–659 10.1016/S0149-7634(01)00060-4 11801290

[B40] Schenberg L. C. Póvoa R. M. F. Costa A. L. P. Caldellas A. V. Tufik S. Bittencourt A. S. (2005). Functional specializations within the tectum defense systems of the rat. Neurosci. Biobehav. Rev. 29, 1279–1298 10.1016/j.neubiorev.2005.05.006 16087233

[B41] Schimitel F. G. de Almeida G. M. Pitol D. N. Armini R. S. Tufik S. Schenberg L. C. (2012). Evidence of a suffocation alarm system within the periaqueductal gray matter of the rat. Neuroscience 200, 59–73 10.1016/j.neuroscience.2011.10.032 22062132

[B42] Schneider C. A. Rasband W. S. Eliceiri K. W. (2012). NIH Image to ImageJ: 25 years of image analysis. Nat. Methods 9, 671–675 10.1038/nmeth.2089 22930834 PMC5554542

[B43] Sessle B. J. Ball G. J. Lucier G. E. (1981). Suppressive influences from periaqueductal gray and nucleus raphe magnus on respiration and related reflex activities and on solitary tract neurons, and effect of naloxone. Brain Res. 216, 145–161 10.1016/0006-8993(81)91283-X 6266582

[B44] Stein M. B. Asmundson G. J. C. (1994). Autonomic function in panic disorder: cardiorespiratory and plasma catecholamine responsivity to multiple challenges of the autonomic nervous system. Biol. Psychiatry 36, 548–558 10.1016/0006-3223(94)90619-X 7827218

[B45] Sun L. Wang H. Hu J. Han J. Matsunami H. Luo M. (2009). Guanylyl cyclase-D in the olfactory CO2 neurons is activated by bicarbonate. Proc. Natl. Acad. Sci. U.S.A. 106, 2041–2046 10.1073/pnas.0812220106 19181845 PMC2644160

[B46] Suvrathan A. Bennur S. Ghosh S. Tomar A. Anilkumar S. Chattarji S. (2014). Stress enhances fear by forming new synapses with greater capacity for long-term potentiation in the amygdala. Philos. Trans. R. Soc. B Biol. Sci. 369, 151–159 10.1098/rstb.2013.0151 24298153 PMC3843883

[B47] Suvrathan A. Tomar A. Chattarji S. (2010). Effects of chronic and acute stress on rat behaviour in the forced-swim test. Stress 13, 533–540 10.3109/10253890.2010.489978 20666651

[B48] Vyas A. Mitra R. Shankaranarayana Rao B. S. Chattarji S. (2002). Chronic stress induces contrasting patterns of dendritic remodeling in hippocampal and amygdaloid neurons. J. Neurosci. 22, 6810–6818 12151561 10.1523/JNEUROSCI.22-15-06810.2002 PMC6758130

[B49] Wang C. Bomberg E. Billington C. Levine A. Kotz C. M. (2007). Brain-derived neurotrophic factor in the hypothalamic paraventricular nucleus increases energy expenditure by elevating metabolic rate. Am. J. Physiol. Regul. Integr. Comp. Physiol. 293, R992–R1002 10.1152/ajpregu.00516.2006 17567712

[B50] Wang Y. Y. Chang R. B. Liman E. R. (2010). TRPA1 Is a component of the nociceptive response to CO2. J. Neurosci. 30, 12958–12963 10.1523/JNEUROSCI.2715-10.2010 20881114 PMC2993877

[B51] Wemmie J. A. (2011). Neurobiology of panic and pH chemosensation in the brain. Dialogues Clin. Neurosci. 13, 475–483 22275852 10.31887/DCNS.2011.13.4/jwemmie PMC3263394

[B52] Wemmie J. A. Askwith C. C. Lamani E. Cassell M. D. Freeman J. H. Welsh M. J. (2003). Acid-sensing ion channel 1 is localized in brain regions with high synaptic density and contributes to fear conditioning. J. Neurosci. 23, 5496–5502 12843249 10.1523/JNEUROSCI.23-13-05496.2003 PMC6741257

[B53] Williams G. Bing C. Cai X. J. Harrold J. A. King P. J. Liu X. H. (2001). The hypothalamus and the control of energy homeostasis. Physiol. Behav. 74, 683–701 10.1016/S0031-9384(01)00612-6 11790431

[B54] Ziemann A. E. Allen J. E. Dahdaleh N. S. Drebot I. I. Coryell M. W. Wunsch A. M. (2009). The amygdala is a chemosensor that detects carbon dioxide and acidosis to elicit fear behavior. Cell 139, 1012–1021 10.1016/j.cell.2009.10.029 19945383 PMC2808123

