# Supplementary material for: Chronic immobilization stress occludes in vivo cortical activation in an animal model of panic induced by carbon dioxide inhalation
Source: Front Behav Neurosci. 2014 Sep 16;8:311. doi: 10.3389/fnbeh.2014.00311 (PMC4165356; doi:10.3389/fnbeh.2014.00311)
Supplement: Supplementary file 1 [file Image1.PDF]

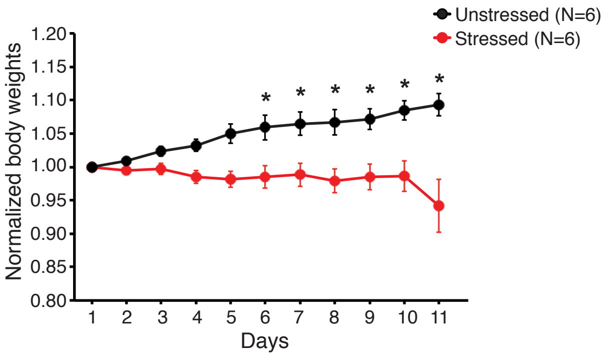

Supplementary Figure S1

Stressed rats do not show body weight gain similar to unstressed rats. Body weights normalized to the body weight on day1 for all the animals. There is a significant difference in normalized body weights of the unstressed and stressed groups from day 6 to day 14 ($p<0.05$, 2-Way repeated measure ANOVA, post hoc Holm-Sidak's test).
