# Supplementary material for: Chronic immobilization stress occludes in vivo cortical activation in an animal model of panic induced by carbon dioxide inhalation
Source: Front Behav Neurosci. 2014 Sep 16;8:311. doi: 10.3389/fnbeh.2014.00311 (PMC4165356; doi:10.3389/fnbeh.2014.00311)
Supplement: Supplementary file 2 [file Image2.PDF]

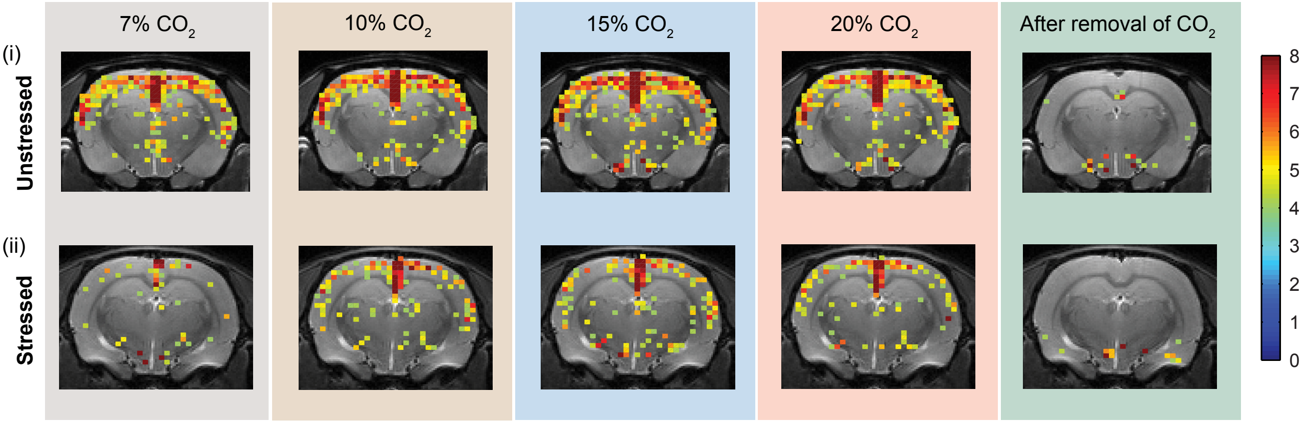

Supplementary Figure S2

CO2 induced dose dependent CBF in stressed and unstressed animals. Activation pattern expressed in terms of z-scores for an unstressed animal (i) and a stressed animal (ii). Only pixels with z-score greater than 3 are represented here.
